# Characterization of the Interferon-Producing Cell in Mice Infected with *Listeria monocytogenes*

**DOI:** 10.1371/journal.ppat.1000355

**Published:** 2009-03-27

**Authors:** Silvia Stockinger, Renate Kastner, Elisabeth Kernbauer, Andreas Pilz, Sandra Westermayer, Benjamin Reutterer, Didier Soulat, Gabriele Stengl, Claus Vogl, Theresa Frenz, Zoe Waibler, Tadatsugu Taniguchi, Thomas Rülicke, Ulrich Kalinke, Mathias Müller, Thomas Decker

**Affiliations:** 1 Max F. Perutz Laboratories, Department of Microbiology and Immunobiology, University of Vienna, Vienna, Austria; 2 Institute of Molecular Pathology, Vienna, Austria; 3 Institute of Animal Breeding and Genetics, University of Veterinary Medicine, Vienna, Austria; 4 Paul-Ehrlich-Institut, Langen, Germany; 5 Graduate School of Medicine and Faculty of Medicine, University of Tokyo, Tokyo, Japan; 6 Biomodels Austria, University of Veterinary Medicine, Vienna, Austria; 7 TWINCORE, Centre for Experimental and Clinical Infection Research, Hanover, Germany; University of Toronto, Canada

## Abstract

Production of type I interferons (IFN-I, mainly IFNα and IFNβ) is a hallmark of innate immune responses to all classes of pathogens. When viral infection spreads to lymphoid organs, the majority of systemic IFN-I is produced by a specialized “interferon-producing cell” (IPC) that has been shown to belong to the lineage of plasmacytoid dendritic cells (pDC). It is unclear whether production of systemic IFN-I is generally attributable to pDC irrespective of the nature of the infecting pathogen. We have addressed this question by studying infections of mice with the intracellular bacterium *Listeria monocytogenes*. Protective innate immunity against this pathogen is weakened by IFN-I activity. In mice infected with *L. monocytogenes*, systemic IFN-I was amplified via IFN-β, the IFN-I receptor (IFNAR), and transcription factor interferon regulatory factor 7 (IRF7), a molecular circuitry usually characteristic of non-pDC producers. Synthesis of serum IFN-I did not require TLR9. In contrast, *in vitro*–differentiated pDC infected with *L. monocytogenes* needed TLR9 to transcribe IFN-I mRNA. Consistent with the assumption that pDC are not the producers of systemic IFN-I, conditional ablation of the IFN-I receptor in mice showed that most systemic IFN-I is produced by myeloid cells. Furthermore, results obtained with FACS-purified splenic cell populations from infected mice confirmed the assumption that a cell type with surface antigens characteristic of macrophages and not of pDC is responsible for bulk IFN-I synthesis. The amount of IFN-I produced in the investigated mouse lines was inversely correlated to the resistance to lethal infection. Based on these data, we propose that the engagement of pDC, the mode of IFN-I mobilization, as well as the shaping of the antimicrobial innate immune response by IFN-I differ between intracellular pathogens.

## Author Summary

Type I Interferons (IFN-I) are cytokines produced by the innate immune system immediately after intrusion of a pathogen. To produce large quantities of IFN-I once an infection is starting to spread throughout the body, the innate immune system employs a specialized “interferon-producing cell” (IPC). In the case of viral infections, IFN-I protect the host organism from rapid viral replication and spread. Conversely, organisms that cannot produce IFN-I are exquisitely sensitive to viral infections. Intriguingly, the opposite has been reported for the pathogen *Listeria monocytogenes*. Like virus, this bacterium replicates within cells of the host organism and stimulates IFN-I synthesis. Unlike virus, however, IFN-I sensitize the infected host to lethal pathology resulting from *L. monocytogenes* infection. In this article, we show that all tested molecules contributing to IFN-I production in Listeria-infected mice are responsible for a corresponding increase in mortality. We address the question of which cell type is responsible for producing vast quantities of IFN-I that can be measured in the serum of mice infected with Listeria. We show that these are not IPC, but rather macrophages, cells specialized to ingest and kill bacteria. We conclude that the engagement of cells for IFN-I production and also the effect of IFN-I on innate immunity is determined by the tropism and lifestyle of a particular pathogen.

## Introduction

Type I interferons (IFN-I) comprise a family of around 20 members that bind a common receptor, the type I IFN receptor (IFNAR) [1]. Immunologically most relevant are IFNβ, with only one member in humans and mice, and the IFNα family with more than ten members. Pattern recognition receptors (PRR) for all classes of microbes are able to stimulate transcription of the IFN-I genes, establishing IFN-I production as a hallmark of innate immune responses to a vast number of viral and nonviral pathogens [2]–[8]. A common property of these receptors is to stimulate the activation of one or more transcription factors of the interferon regulatory factor (IRF) family, most importantly IRF3 and IRF7 [9].

Cells producing IFN-I employ different molecular strategies to induce synthesis of the cytokines. A feed-forward amplification mode is characterized by IRF3-dependent IFNβ production that predominates the early phase of infection. In a subsequent phase signaling of the IFNβ through the IFNAR stimulates activation of the transcription factor ISGF3 consisting of a STAT1/STAT2 heterodimer in conjunction with IRF9 [10]. This transcriptional complex activates the IRF7 gene promoter and initiates expression of the IRF7 mRNA. Finally, IRF7 is instrumental in transcribing multiple IFNα genes in addition to IFNβ [11]. An alternative way of stimulating IFN-I was described for virus-infected or toll-like receptor (TLR) ligand-stimulated plasmacytoid dendritic cells and, under certain conditions, myeloid dendritic cells (pDC and mDC, respectively) [12]–[14]. In these cells, the IFN-I genes are targeted by a pathway originating from endosomal TLRs (TLR 7 and TLR 9) that assemble a signalosome including MyD88 and IRF7 together with other IRFs such as IRF1 or IRF5 [15]–[20]. In pDC the TLR signal is rapidly relayed to IRF7, which is available for immediate stimulation of all IFN-I promoters. Thus, pDC produce vast quantities of IFN-I in response to infection with viruses and are generally referred to as interferon-producing cells (IPC).

*Listeria monocytogenes* is a Gram-positive, food-borne bacterial pathogen [21]. Major sites of Listeria replication during systemic infection of mammals are liver and spleen. Infection is exacerbated by the activity of IFN-I, shown convincingly by the increased resistance of IFNAR or IRF3-deficient mice to lethal infection [22]–[24]. Several explanations for this IFN-I effect have been provided, including decreases in TNF production or an enhancement of macrophage death [22]–[25]. Moreover, IFN-I were shown to sensitize cytotoxic T lymphocytes to the lytic action of LLO [24]. Enhanced lymphocyte killing and IL-10-dependent suppression of innate immunity as a result of phagocytic uptake of apoptotic cells increase the severity of infection-borne pathological effects [26].

*L. monocytogenes* is endowed with the ability to infect the cytoplasm of cells in the host organism [27]. This ability results from the endo/phagosome-disrupting activity of the major *L. monocytogenes* virulence factor, the Listeriolysin O (LLO), a bacterial cholesterol-dependent hemolysin [28]. A common property of *L. monocytogenes* and viruses is that recognition and signaling occur through both membrane-associated and cytosolic PRR [3]. In contrast to viruses, the cytosolic *L. monocytogenes* receptors mediating IFN-I induction have not been identified. In *L. monocytogenes*-infected macrophages, IFN-I synthesis occurs via feed-forward amplification [29]. The exact pathway and cell type responsible for IFN-I production during infection of mice with *L. monocytogenes* have not been examined. Considering the fact that IFN-I enhance the adverse effects of the early, innate immune response to *L. monocytogenes* [22]–[24], it is of major importance to understand their mobilization and mode of action.

In this study we sought to identify the essential IFN-I-producing cells during infection with *L. monocytogenes*. We used sorted splenic cell populations and multiple mouse models with defects in the IFN-I production machinery or with tissue-specific disruption of the *ifnar1* gene to decipher the pathways and cells required for both IFNβ and IFNα production. Our results show that in contrast to viral infection models, pDC are not the source of IFN-I in response to *L. monocytogenes*. Rather, the cytokines are produced via feed-forward amplification by a splenic cell with cell surface markers characteristic of macrophages. We also show for the first time that IFNβ and IRF7 make strong contributions to the harmful action of IFN-I in *L. monocytogenes* infection because the lower levels of IFN-I production in these mouse lines were correlated with decreased susceptibility to lethal infection.

## Results

### Prerequisites for IFN-I production in mice and cells infected with *L. monocytogenes*

**Figure 1:**
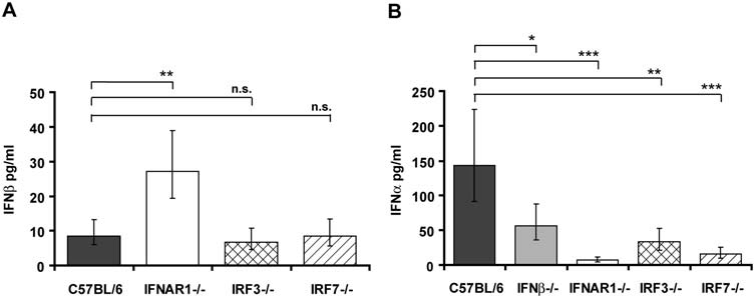
Serum levels of IFN-I in mice deficient in components of the IFN-I signaling pathway. Mice of the indicated genotypes were injected intraperitoneally (i.p.) with 5×10^6^ *L. monocytogenes* (C57BL/6 n = 25, IFNβ−/− n = 25, IFNAR1−/− n = 24, IRF3−/− n = 25, IRF7−/− n = 20) or PBS as a control (data not shown). After 24 h, serum was collected and ELISAs for IFNβ (A) and IFNα (B) were performed. The data presented here are a summary of several individual experiments with groups of four to five infected mice per genotype. Data representing IFNβ (A) and IFNα (B) concentrations were log-transformed (after adding one) to achieve approximate normality. Linear models with genotype and experiment as fixed effects were fitted using SPSS. Means plus/minus standard errors of wt and mutant genotypes are plotted (after back-transformation). Significant values are indicated by: n.s. not significant p>0.05, * p≤0.05, ** p≤0.01, *** p≤0.001.

**Figure 2:**
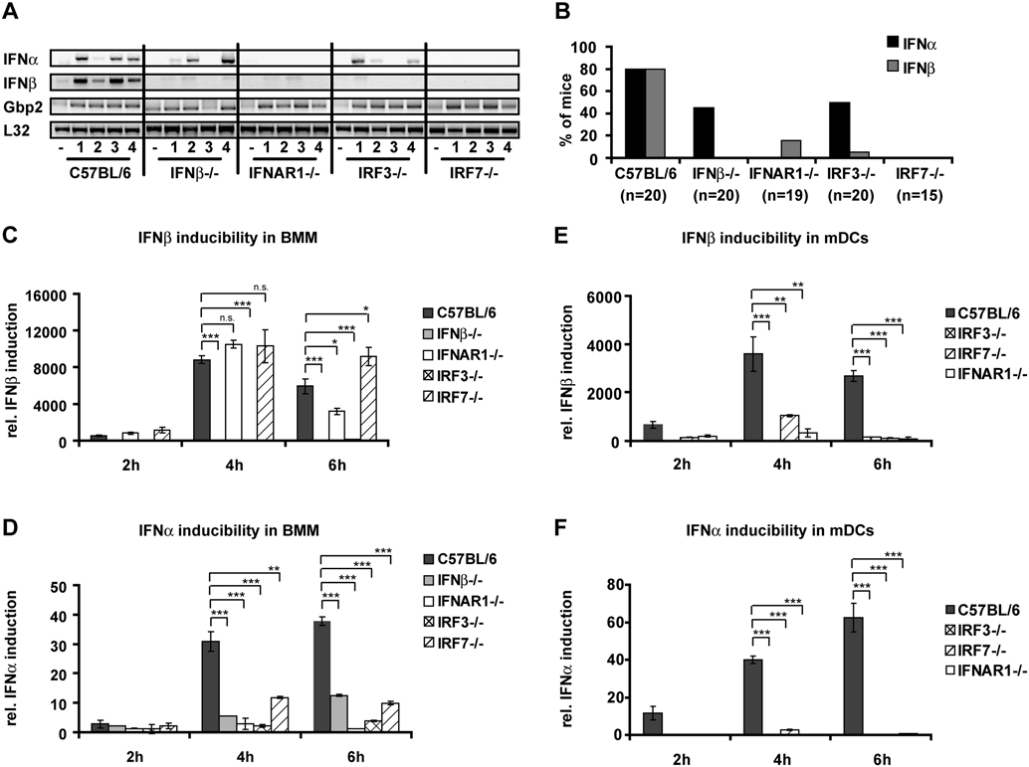
Prerequisites for IFN-I production in infected spleens and *in vitro*–generated BMM and mDCs. Mice of the indicated genotypes were injected i.p. with 5×10^6^ *L. monocytogenes* or PBS as a control. After 24 h of infection, mice were killed and spleens and livers isolated. (A) RNA was extracted from spleens of four infected mice per genotype (1–4) or from mice injected with PBS (−), and RT-PCR for the indicated genes was performed. As a normalization control, the housekeeping gene L32 was measured. (B) Summary of experiments performed with mice of the indicated genotypes. In total, 20 C57BL/6, 20 IFNβ−/−, 19 IFNAR1−/−, 20 IRF3−/−, and 15 IRF7−/− mice were analysed for IFN-I expression and expression of Gbp2. The graph shows the percentage of mice of the respective genotypes showing detectable IFNα (black) or IFNβ (grey) expression after infection with 5×10^6^ *L. monocytogenes* for 24 h. (C–F) BMM (C,D) or mDC (E,F) of the indicated genotypes were infected with *L. monocytogenes* at a MOI of 10. At the indicated time points, total RNA was prepared. The isolated RNA was reverse-transcribed and induction of the IFNβ (C,E) or IFNα genes (D,F) was measured by real-time PCR. For normalization to a housekeeping gene, GAPDH was measured.

**Figure 3:**
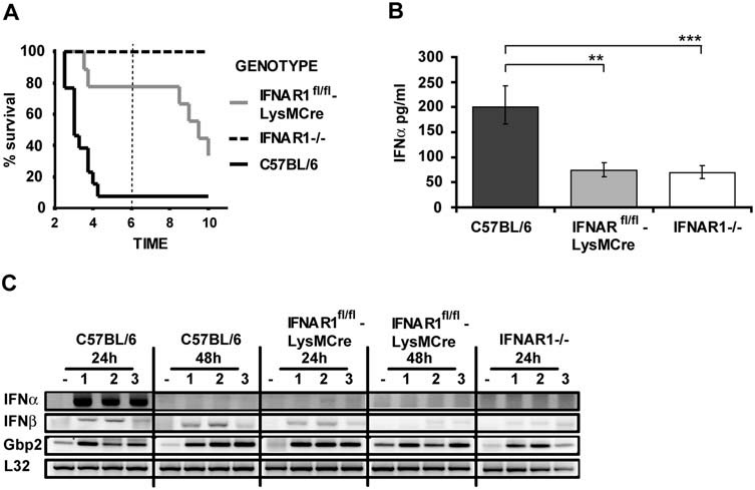
Myeloid cells strongly contribute to IFN-I production. (A) C57BL/6 wt (n = 13), IFNAR1−/− (n = 4), and mice with LysM-Cre-mediated deletion of IFNAR1 (LysMCre-IFNAR1^fl/fl^, n = 9) were infected with 5×10^6^ *L. monocytogenes*, and survival was monitored for ten days. (B) Mice of the indicated genotypes were infected with 5×10^6^ *L. monocytogenes* (seven mice per group) or injected with PBS (two mice per group). After 24 h, serum was collected and ELISA was performed for IFNβ (data not shown) and IFNα levels. Data representing IFNα concentrations were log-transformed to achieve approximate normality. Linear models with genotype as fixed effect were fitted using SPSS. Means plus/minus standard errors of wt and mutant genotypes are plotted (after back-transformation). Significant values are indicated by ** p≤0.01, *** p≤0.001. (C) Mice of the indicated genotypes were infected with 5×10^6^ *L. monocytogenes* (1–3) or injected with PBS (−). After 24 h and 48 h, spleens were isolated and RNA was extracted. The RNA was reverse-transcribed and subjected to PCR for the indicated genes. As a normalization control, the housekeeping gene L32 was measured.

Knock-out mice were used to study pathways contributing to increase of IFNβ and IFNα in the serum of mice infected with *L. monocytogenes*. We established 24 h post intraperitoneal (i.p.) injection of bacteria as the point of maximum IFN-I induction in preliminary experiments (data not shown); therefore, IFNβ and IFNα were measured 24 h after infection with *L. monocytogenes* in subsequent experiments. Serum levels of IFNβ were generally at the detection limit of the ELISA and differences between wt and the IRF3−/− or IRF7−/− genotypes could not be reliably determined. By contrast, statistically significant differences were observed comparing wt and IFNAR1−/− animals (Figure 1A). This finding most likely reflects the importance of the IFNAR for IFNβ clearance. Increased accumulation of the cytokine in the blood of IFNAR−/− mice is therefore not in contradiction to subsequent findings showing that a fraction of IFNβ production occurs via feed-forward amplification through the IFNAR (e.g. Figures 2 and 3). Serum of infected wt mice contained higher levels of IFNα than IFNβ. IFNα species were reduced in absence of IFNβ, IRF3 or IRF7 (Figure 1B). Serum IFNα was virtually absent in mice lacking IFNAR1. Collectively the data show that the levels of serum IFNα critically depend on ‘early’ IFNβ and on IFN-I signaling, hence feed-forward amplification. Bacterial loads in liver and spleen after 24 h of infection were virtually identical between wt and IFNAR−/− mice (Figure S1). Therefore, differences in IFN-I production did not result from lower numbers of infecting bacteria.

The spleen is a major target organ of *L. monocytogenes*, irrespective of the route of infection by intragastric, intraperitoneal or intravenous application [21],[30]. Therefore, we tested both IFN-I production and response in spleens following infection with *L. monocytogenes* (Figure 2A and 2B, and Figure S2A). Production of both IFNβ and IFNα mRNA, assayed by RT-PCR (Figure 2A and 2B), was observed in wt animals. IFNβ mRNA synthesis required both IRF3 and IRF7 and, surprisingly, the IFNAR. Splenic IFNα production was reduced in absence of IRF3 or IFNβ and even more affected by the lack of IRF7 or the IFNAR. A representative analysis of four mice/genotype is shown in Figure 2A, whereas Figure 2B summarizes all experiments to show how many mice of each genotype produced IFN-I and account for individual responses to Listeria infection.

STAT2 is a subunit of the ISGF3 transcription factor complex and its tyrosine phosphorylation is a hallmark of IFN-I-treated cells [10]. Phosphorylated STAT2 was absent in IFNAR1−/− spleens, reduced slightly in absence of IFNβ and IRF3, and reduced strongly in absence of IRF7 (Figure S2A). STAT1 tyrosine phosphorylation occurs in response to both IFN-I and IFNγ [10]. Likewise, the STAT target gene Gbp2 (Figure 2A) responds to both IFN types. Both STAT1 tyrosine phosphorylation and Gbp2 expression occurred at levels similar to wt in all genotypes including IFNAR1−/− (Figure S2A). In agreement with this, IFNγ mRNA levels were equal to wildtype in all investigated genotypes (data not shown). Together the data show that the molecular requirements for splenic IFN-I production are very similar to those found for serum IFNα production. In both cases signaling through IFNAR and upregulation of IRF7 expression are of critical importance and indicate feed-forward amplification.

In bone-marrow-derived macrophages (BMM), *L. monocytogenes* stimulates IFNβ synthesis through the IRF3 pathway independently of TLR9 and MyD88 [29],[31]. Requirements for IFN-I production were further investigated in BMM and mDC infected with *L. monocytogenes*. IRF7 or IFNAR-deficiency had no effect on BMM IFNβ production (Figure 2C). Unlike IFNβ, IFNα production by infected BMM was dependent on IRF3, IRF7, IFNβ and the IFNAR (Figure 2D). In case of IRF7-deficiency, residual IFN-I still caused unimpaired tyrosine phosphorylation of STAT2 (Figure S2B). In mDC, surprisingly, both IFNβ and IFNα production strictly required IRF3, IRF7 and IFNAR1 (Figure 2E and 2F).

### Prerequisites for the increase in resistance to lethal infection caused by IFN-I

**Figure 4:**
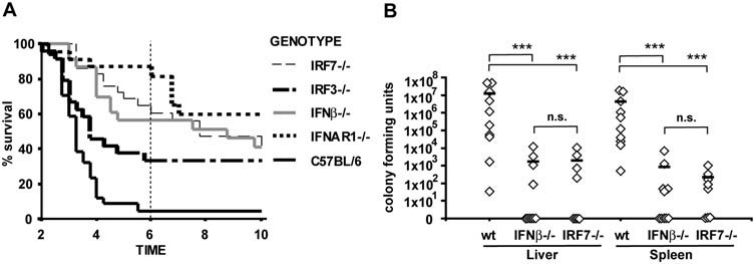
Prerequisites for the increase in resistance to lethal infection caused by IFN-I. (A) Mice of the indicated genotypes were infected with 5×10^6^ *L. monocytogenes*, and survival was monitored for ten days. The data presented here are a summary of several individual experiments with groups of 4–6 mice per genotype and treatment. Total number of mice analysed are: C57BL/6 wt n = 34, IFNAR1−/− n = 23, IFNβ−/− n = 23, IRF3−/− n = 24, IRF7−/− n = 29. (B) Groups of ten (C57BL/6 wt and IFNβ−/−) or eight (IRF7−/−) mice were infected with 1×10^6^ *L. monocytogenes*. After three days, mice were killed and the *L. monocytogenes* titre was determined in the liver and spleen and presented as CFU. Data were log-transformed to achieve approximate normality. Linear models with genotype as fixed effect were fitted using SPSS. Significant values are indicated by: n.s. not significant p>0.05, *** p≤0.001.

To investigate the contribution of IFNβ and IRF7 to the IFN-I-dependent increase in mortality, we monitored the survival of animals infected with *L. monocytogenes*. In accordance with previous findings [22]–[24], IFNAR deficiency caused a strong resistance to lethal infection, particularly during the innate phase of the anti-Listeria immune response (up to day 6, Figure 4A). By comparison, the increase caused by IFNβ deficiency was less pronounced. Lack of IRF7 had a greater impact on survival than absence of IRF3. This difference became smaller if survival was monitored beyond the period of the innate immune response (up to day 10). Lower pathogen burdens in liver and spleens from IRF7 or IFNβ-deficient mice reflected the increase in survival on day 3 (Figure 4B). Similar findings have been reported for IFNAR1 and IRF3-deficient mice [22]–[24]. Since differences in the bacterial load between wt animals and those with defects in IFN-I synthesis and/or response are not present 24 h after infection (Figure S1) the inhibitory effect of IFN-I on bacterial clearance must develop between day 1 and 3 post infection. Together, the data emphasize the importance of the early IFNAR, IFNβ and IRF7-mediated amplification of IFN-I production for adverse IFN-I action during the early, innate immune response.

### Myeloid cells contribute to IFN-I production and the IFN-I–dependent sensitization of mice to lethal infection with *L. monocytogenes*

To examine the cell type(s) important for IFN-I production and response during *L. monocytogenes* infection, we made use of tissue-specific IFNAR ablation. The contribution of myeloid cells to IFN-I effects was tested by mating mice with a floxed *ifnar1* allele [32] with LysM-Cre mice [33]. Using this technology, a high degree of floxed allele conversion is obtained in macrophages and neutrophils, whereas it is inefficient in splenic DC [33]. In accordance with these observations, IFNAR1 expression in IFNAR^fl/fl^-LysM-Cre mice was strongly reduced in CD11b^+^ cells that include macrophages, but similar to wt on CD11c^+^ cells that include myeloid and plasmacytoid DC (Figure S3). Absence of the IFNAR in myeloid cells caused a highly significant increase in the survival of Listeria-infected mice during the innate phase of the immune response up to day 6 (Figure 3A). In this period we observed 100% survival in mice with complete IFNAR1 deletion and 80% survival in mice with myeloid IFNAR1 deletion. At later times the survival curves diverged further, most likely due to a contribution of non-myeloid cells to the lethal outcome of infection. The increase in survival compared to wt mice could result from an important role of myeloid cells in IFN-I production. Alternatively, the myeloid response to IFN-I might directly contribute to the cytokines' detrimental action. To distinguish between these possibilities, serum IFN–I was compared in wt mice and mice lacking the IFNAR either on all or only on myeloid cells. This experiment revealed a major contribution of myeloid cells to serum IFNα by IFNAR-dependent feed-forward amplification (Figure 3B). Differences in the low level of serum IFNβ were not statistically significant between wt mice and those disrupted for the myeloid *ifnar1* gene (data not shown).

Analysis of splenic IFN-I production and response in mice lacking the myeloid IFNAR showed that IFNα mRNA was completely absent (Figure 3C). Residual IFNβ mRNA was present, but the induction was reduced and more transient (compare 24 h and 48 h time points). No difference between genotypes was observed for the induction of Gbp2.

### IFN-I production and response in *L. monocytogenes*–infected hosts is independent of the TLR9 pathway

**Figure 5:**
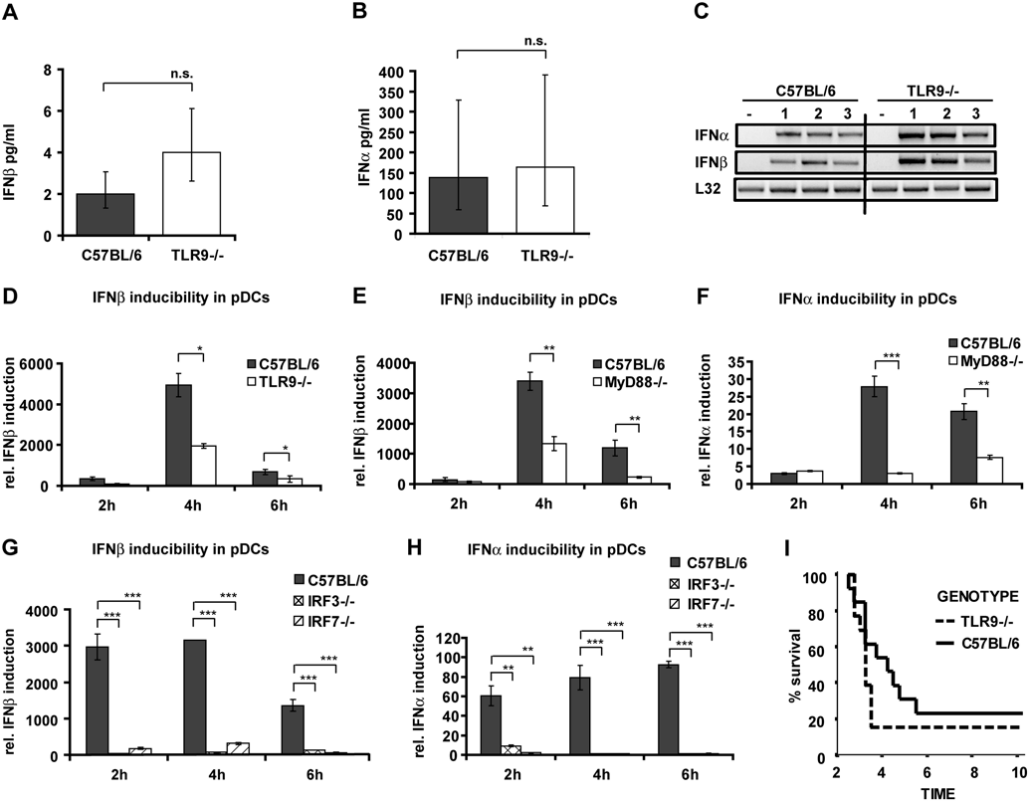
The role of TLR9 in mice and cells infected with *L. monocytogenes*. (A–C) C57BL/6 wt or TLR9−/− mice were injected i.p. with 5×10^6^ *L. monocytogenes* (four mice per genotype) or PBS as a control (data not shown). After 24 h of infection, serum was collected and ELISAs for IFNβ (A) and IFNα (B) were performed. Data representing IFNβ (A) and IFNα (B) concentrations were log-transformed (after adding one) to achieve approximate normality. Linear models with genotype as fixed effect were fitted using SPSS. Means plus/minus standard errors of wt and mutant genotypes are plotted (after back-transformation), n.s.: not significant (p>0.05). (C) After 24 h of infection, mice were killed and spleens isolated. RNA was extracted from spleens of three infected mice per genotype (1–3) or from mice injected with PBS (−), and RT-PCR for the indicated genes was performed. As a normalization control, the housekeeping gene L32 was measured. (D–H) pDCs of the indicated genotypes were infected with *L. monocytogenes* at a MOI of 10. At the indicated time points, total RNA was prepared. The isolated RNA was reverse-transcribed, and induction of the IFNβ (D,E,G) or IFNα (F,H) genes was measured by real-time PCR. For normalization to a housekeeping gene, GAPDH was measured. (I) C57BL/6 wt or TLR9−/− mice (13 mice per genotype, data represent a summary of two experiments) were injected i.p. with 5×10^6^ *L. monocytogenes*. Survival was monitored for ten days.

The data collected so far suggest that the majority of IFN-I is produced by a splenic cell type that employs an IFNβ/IFNAR/IRF7 pathway for feed-forward amplification of IFNα synthesis. This profile does not match IFN-I production by pDC where expression of IFN-I genes is regulated by a pathway originating from endosomal TLRs [34]. To further exclude the relevance of this pathway for IFN-I production during Listeria infection, TLR9-deficient mice were analyzed. The data summarized in Figure 5 show that neither serum IFNβ or IFNα (Figure 5A and 5B), nor splenic IFN-I mRNA synthesis or response were reduced in the absence of TLR9 (Figure 5C, Figure S4A). By contrast, production of IFN-I mRNA in bone-marrow-derived pDCs exposed to *L. monocytogenes* showed strong (IFNβ) or absolute (IFNα) dependence on TLR9 and/or MyD88 (Figure 5D–5F). Both IFNβ and IFNα mRNA synthesis were strongly affected by IRF7 deficiency and, surprisingly, also the absence of IRF3 (Figure 5G and 5H). Unlike pDC, mDC produced IFNβ independently of the TLR9 pathway, resembling macrophages in this regard (Figure S4B). Consistent with IFN-I production (Figure 5A–5C), the absence of TLR9 did not increase survival following infection with *L. monocytogenes* (Figure 5I). Rather, TLR9 deficiency slightly enhanced the lethality of *L. monocytogenes* infection, suggesting that the TLR9 pathway plays a protective role.

### Cells with a surface profile characteristic of macrophages are the IFNα producers in *L. monocytogenes*–infected spleens

**Figure 6:**
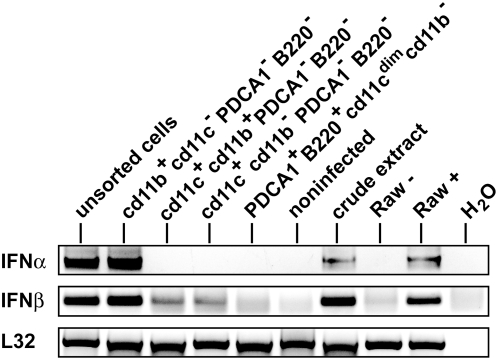
Macrophages and not pDC are the IFN-I–producing cells after *L. monocytogenes* infection. C57Bl/6 wt mice (n = 15) were infected with 5×10^6^ *L. monocytogenes*. After 24 h, mice were killed, splenic cells were isolated, labelled with antibodies against CD11b (FITC-conjugated), CD11c (APC), PDCA1 (PE-Cy5), and B220 (PE-Cy7), and subjected to FACS sorting. RNA of the isolated cell populations was extracted, reverse-transcribed, and subjected to PCR for the indicated genes. Unsorted cells and whole spleen extracts from infected spleens (crude extract) as well as uninfected spleens were used as controls. As a positive control for the PCR reactions, Raw264.7 macrophages were infected (+) or not (−) with *L. monocytogenes* for 8 h at a MOI of 10. As a negative control, H_2_O was used instead of template cDNA. As a normalization control, the housekeeping gene L32 was measured.

To further rule out pDC as major producers of IFN-I during Listeria infection of mice and to confirm a prominent role of myeloid cells, we purified cell populations from *L. monocytogenes* infected spleens by FACS and measured IFN-I production by RT-PCR. Figure 6 clearly shows that cells with a phenotypic profile of pDC (PDCA1^+^B220^+^CD11c^dim^CD11b^−^) fail to produce IFN-I in response to *L. monocytogenes* infection. The CD11c^hi^ population (either CD11b^+^PDCA1^−^B220^−^ or CD11b^−^PDCA1^−^B220^−^) including typical mDCs did not express detectable IFNα mRNA, whereas these cells produced minor amounts of IFNβ mRNA. In contrast, CD11b^+^ cells not expressing CD11c, PDCA1 or B220, a profile characterizing macrophages, expressed high levels of both IFNα and IFNβ mRNA. Macrophages, therefore, are most likely the predominant IFN-I producers in Listeria-infected mice.

## Discussion

Using mice and cells ablated for genes involved in IFN-I synthesis, we established a molecular profile of the cell type(s) producing IFN-I upon infection with *L. monocytogenes* and correlated IFN-I production in the spleen and serum with the survival of infected hosts. Clear results emerging from our studies are that I) the TLR9/MyD88 pathway, a hallmark of IFN-I synthesis by pDC, does not significantly contribute to splenic (local) or systemic IFN-I production; II) consistently, feed-forward amplification requiring signaling through the IFNAR is of great importance and III) the main IFN-I producers are cells with characteristics typical of splenic macrophages. Finally and IV), IFNβ and IRF7 contribute to the enhancement of lethal infection by IFN-I. Reduction in IFN-I production that resulted from IRF3, IRF7, IFNβ or IFNAR deficiency by and large reflected the increase in resistance to lethal infection. Examination of pathogen organ loads revealed that the bacterial growth-promoting, hence lethality-enhancing activity of IFN-I develops between day 1 and 3 post infection.

Our studies focused on the early, innate phase of infection up to about day 6. IFN-I may additionally influence the ensuing adaptive immune response and, as suggested by recent studies, this effect may not be primarily adverse as in the innate response [35]–[37]. Diverse IFN-I effects on adaptive and innate immunity most likely explain that the relative increases in resistance to infection caused by the knock-out mice used in our study differ to some degree when analyzed at day 6 or day 10 after infection (Figure 4). Furthermore, the survival curve of mice with myeloid cell-specific IFNAR1 ablation suggests that cells outside this cell compartment influence the survival of Listeria infection particularly after the innate phase of the immune response.

Two recent studies closely examining the innate response to Listeria in infected spleens revealed a highly complex process requiring CD11c^+^ DC for the movement of Listeria to the white pulp and to initiate a concerted response involving macrophages, TIP-DC, NK cells and neutrophils [38],[39]. Our study shows that the TLR9 pathway was not engaged by *L. monocytogenes* for IFN-I synthesis in mice. By contrast, data with pDC-enriched bone marrow cultures show that Listeria can present a TLR9 ligand and stimulate this cell type for IFN-I production. This finding strongly implies that pDC do not contribute to IFN-I production during *L. monocytogenes* infection of mice, in spite of high bacterial loads in lymphoid organs that harbor this cell type. Although TLR9/MyD88-independent IFN-I synthesis has also been observed in mice infected with viruses [40]–[42], several reports suggest that the engagement of the pDC/TLR system for IFN-I production occurs when viruses gain access to lymphoid tissue either directly or because the pathogen breaks the initial barrier of infection formed by macrophages and/or epithelial cells [34], [41]–[43]. For example, lung infection with Newcastle disease virus led to IFNα production by alveolar macrophages, whereas systemic infection engaged both mDC and pDC for IFNα production [41]. Splenic mDC are reportedly major IFN-I producers during infection with Adenovirus, underscoring pathogen specificity in the choice of IPC and defying a general role of pDCs as producers of systemic IFN-I upon viral infection [44]. Under steady state conditions, pDC are mainly found in peripheral blood and lymphoid organs [13]. A recent report by Tam and Wick demonstrated expansion and activation of cells phenotypically resembling pDC in lymphoid tissue infected with *L. monocytogenes* [45]. The cells expressed activation antigens, but, consistent with our results, did not produce cytokines. Together, the data suggest that the ability of *L. monocytogenes* to stimulate pDC expansion occurs without direct contact and may be caused by cytokines released from infected cells. Unlike virus, *L. monocytogenes* appears to be unable to efficiently gain access to pDC even at lethal doses of infection when lymphoid organs are heavily infected. Direct contact may be prevented by an efficient containment of the pathogen in cells that are more readily infected than pDC. Owing to their phagocytic potential, splenic macrophages activated by NK cell-derived IFNγ, might clear Listeria before they reach pDC. It will be interesting to determine whether the choice of IPC determines the IFN-I effect on infection and whether macrophages as IFN-I producers are related to fatal outcome as in the case of *L. monocytogenes*. In this regard it will be important to examine IPCs in murine infection models such as *S. pneumoniae*, where the host benefits from the production of IFN-I [46].

IFNα production in BMM via feed-forward amplification required IRF3-dependent IFNβ production, signaling through IFNAR1 and IRF7. This resembled the molecular mechanism observed for splenic or systemic IFNα production after *in vivo* infection. To confirm the assumption that cells different from pDC are important IFNα producers *in vivo* we used IFNAR^fl/fl^-LysM-Cre mice and demonstrated that IFNα synthesis critically depends on the presence of IFNAR1 on myeloid cells. Consistently, cells expressing surface markers characteristic of macrophages express both IFNβ and IFNα mRNA when purified from infected spleens. By contrast, this was not observed for the cell populations that include mDC and pDC. Thus, macrophages are the most likely IFNα producer cells after *L. monocytogenes* infection *in vivo*. An additional contribution of neutrophils cannot be ruled out entirely. However, little is known about the presence and abundance of this cell type in the spleen early after Listeria infection and it is similarly unclear whether neutrophils produce IFN-I in response to infection.

Whereas IRF3 was also critically involved in IFNβ production by bone marrow-derived macrophages, the IFNAR or IRF7 were not required. This contrasts the more important role of IRF7 for IFNβ synthesis in the *L. monocytogenes* infected spleen. Unlike bone marrow macrophages, much of splenic IFNβ resulted from an IFNAR/IRF7-dependent amplification loop because IFNAR−/− spleens expressed strongly reduced amounts of IFNβ mRNA. This may reflect a contribution of splenic mDC to IFNβ production. This cell type required IRF3 as well as IFNAR/IRF7 for IFNβ synthesis also when grown from mouse bone marrow. We assume that mDC produce low amounts of IFNβ immediately after Listeria infection in an IRF3-dependent manner. This initiates the feed-forward amplification loop causing synthesis of the majority of both IFNβ and of IFNα. This model, while consistent with the data, fails to provide a satisfactory explanation for the obvious difference between bone marrow-derived macrophages and mDC regarding the significance of IFNAR1/IRF7 for IFNβ synthesis. Possibly the much larger rate of cytoplasmic infection of macrophages drives IRF3-dependent IFNβ synthesis much more efficiently. A deeper understanding of cell type differences in the mode of IFN-I production is needed to provide ultimate clarity regarding this point. The assumption that splenic mDC participate in IFNβ production via IFNAR and IRF7 in infected mice is supported by the presence of mRNA for the cytokine in CD11c^+^/CD11b^+^ cells purified from infected mice. Nonetheless, a larger amount of IFNβ mRNA was detected in purified splenic macrophages, suggesting a major contribution of these cells to total IFNβ production. At the same time, a large fraction of splenic IFNβ requires feed-forward amplification and, judging from our findings with BMM, this appears to contrast the assumption of macrophages being major producers. A simple explanation for this discrepancy might be an intrinsic difference between splenic macrophages and BMM regarding feed-forward amplification of IFNβ production. Alternatively, the IFNAR/IRF7 requirement of splenic macrophages may derive from the fact that feed-forward amplification results from paracrine, not autocrine IFN-I priming. Under this assumption IFN-I is released by infected macrophages within or outside the spleen to prime uninfected splenic macrophages. Signaling through the IFNAR will result in IRF7 expression and the transcription factor is available for both IFNβ and IFNα production once primed cells become infected by Listeria. By contrast BMM are synchronously infected in culture and will immediately produce IFNβ in absence of IRF7 expression, employing exclusively IRF3 instead.

In summary our results provide answers to the question how the IFN-I system is deployed by an intracellular bacterial pathogen. Both, the observations made with cultured cells and our studies in mice, emphasize that molecular mechanisms governing IFN-I synthesis and response strongly depend on the pathogen as well as the host cell type. Deciphering the importance of these differences for various routes of infection and for diverse types of pathogens remains a challenging task for future research.

## Materials and Methods

### Ethics Statement

All animal experiments were discussed and approved by the University of Veterinary Medicine, Vienna institutional ethics committee and carried out in accordance with protocols approved by the Austrian law (GZ 680 205/67-BrGt/2003).

### Bacteria

*Listeria monocytogenes* LO28 [47] were grown in brain heart infusion (BHI) (Difco) broth. Concentrations of bacteria were determined by measurement at OD_600_ and confirmed by plating serial dilutions onto BHI or Oxford agar (Merck) plates.

### Mice

All mice were on a C57BL/6 background and housed under specific pathogen-free conditions according to FELASA guidelines [48].

### Cells

Raw264.7 macrophages were cultured in DMEM (Gibco, Invitrogen) supplemented with 10% FCS (Gibco, Invitrogen). Bone marrow was isolated from femurs of 6–8 week old mice. For differentiation of BMM, cells were grown in DMEM (Gibco, Invitrogen) in the presence of 10% FCS (Gibco, Invitrogen) and L-cell derived CSF-1 as described [49]. The cultures contained >99% F4/80^+^ cells. mDCs were obtained by culture of bone marrow in DMEM (Gibco, Invitrogen) supplemented with 10% FCS (Gibco, Invitrogen) and X-6310 derived GM-CSF as described [50]. mDC cultures contained virtually no F4/80+ cells and the purity of CD11c^+^/CD11b^+^ cells was between 60 and 70%. pDCs were obtained by culture of bone marrow in DMEM (Gibco, Invitrogen) supplemented with 50 ng/ml of Flt-3-L for 6 days. The purity of CD11c^dim^/B220^+^ pDCs was between 60 and 70%. pDC cultures contained no F4/80^+^ cells.

### Infection of cells

Raw264.7 macrophages, primary BMM, mDCs and pDCs were infected with *L. monocytogenes* (derived from overnight culture) at a multiplicity of infection (MOI) of 10 and incubated for 60 min at 37°C in a humidified CO_2_ atmosphere. Extracellular bacteria were subsequently killed by exchanging medium to gentamicin-containing medium (final concentration 50 µg/ml). After another 60 min, medium was changed to medium containing 10 µg/ml gentamicin.

### Infection of mice

*Listeria monocytogenes* was grown in BHI broth (Difco) to late logarithmic phase (OD_600_ 0.8), pelleted and resuspended in BHI containing 20% glycerol. Aliquots were shock-frozen and stored at −80°C. The concentration of *L. monocytogenes* aliquots was quantified by plating serial dilutions onto Oxford agar plates (Merck). For infection, the bacteria were thawn on ice, washed with PBS for three times and diluted in PBS (endotoxin-free, Sigma) to the appropriate concentrations. 500 µl of the bacterial suspension were injected into the peritoneum of 10- to 12-week-old mice. For the experiments shown, survival of infected mice was monitored for 10 days. For determination of the bacterial load, mice were killed at the indicated time points after infection and spleens and livers were homogenized in PBS. Serial dilutions of homogenates were plated on Oxford agar plates and colonies were counted after growth at 37°C for 24–36 h. For RNA and protein extraction from spleen and for sorting of splenic cells, mice were killed after 24 h of infection and spleens were isolated.

### ELISA

Mice were infected intraperitoneally with *L. monocytogenes* or injected with PBS (Sigma). Sera were collected after 24 h of infection and levels of IFNβ and IFNα were determined by ELISA according to the manufacturer's instructions (PBL InterferonSource).

### Fluorescence-activated cell sorting (FACS)

Spleens were digested in 1 mg/ml collagenase (Roche) for 10 min and homogenized using a cell strainer. The cell suspension was labelled with the following antibodies: CD11b−FITC (Becton Dickinson), CD11c-APC (Becton Dickinson), B220-PE-Cy7 (Beckton Dickinson) and PDCA1-PE-Cy5 (Miltenyi Biotec). Four different cell populations were sorted by FACS: PCDA1^+^B220^+^CD11c^dim^ (pDC), CD11b^+^CD11c^−^B220^−^PDCA1^−^ (macrophages), CD11b^+^CD11c^+^B220^−^PDCA1^−^ and CD11b^−^CD11c^+^B220^−^PDCA1^−^ (myeloid dendritic cells).

### RNA extraction and PCR

RNA extraction from cells, reverse transcription and real-time PCR was performed as already described [29]. Data obtained by real-time PCR were analysed using SPSS and a Student's t-Test. Significant values are indicated by: n.s. not significant p>0.05, * p≤0.05, ** p≤0.01, *** p≤0.001.

Primers for real-time PCR were as described, except for IFNα: Probe: panIFNα: 5′-(6-Fam) AG+AA+GAA+A+C+AC+AG+CC (BHQ1)-3′ (+indicating LNA (Locked Nucleic Acid) nucleotides; Proligo); panIFNα-for: 5′-CCACAGGATCACTGTGTACCTGAGA-3′; panIFNα-rev: 5′-CTGATCACCTCCCAGGCACAG-3′

Spleens were isolated from mice infected with *L. monocytogenes* or injected with PBS for 24 h, shock-frozen in liquid nitrogen and stored at −80°C. For RNA extraction, 20 mg of frozen tissue was homogenized in lysis buffer RA1 (NucleoSpin RNAII kit, Macherey-Nagel) with the Precellys 24 homogenizer (Peqlab) at 6000 rpm for 30 sec. Subsequent isolation of total RNA and cDNA synthesis was performed as described [29]. Primers used for PCR were as follows: panIFNα-for-5′-ATGGCTAG(A/G)CTCTGTGCTTTCCT-3′; panIFNα-rev-5′-AGGGCTCTCCAGA(T/C)TTCTGCTCTG-3′; IFNβ-for 5′-CATCAACTATAAGCAGCTCCA-3′;

 IFNβ-rev 5′-TTCAAGTGGAGAGCAGTTGAG-3′; Gbp2-for-5′-TGCTAAACTTCGGGAACAGG-3′; Gbp2-rev-5′-GAGCTTGGCAGAGAGGTTTG-3′;

 L32-for 5′-ATTAAGCGAAACTGGCGGAAACCC-3′; *L32-rev 5′- TTTCTTCGCTGCGTAGCCTGG-3′*.

Supplementary Materials and Methods are presented in Text S1.

## Supporting Information

Figure S1 Groups of 9 C57BL/6 wt or IFNAR1−/− mice were infected with 5×10^6^ *L. monocytogenes*. After 24 h of infection, mice were killed and the *L. monocytogenes* titre was determined in the liver and spleen and presented as CFU. Data were log transformed to achieve approximate normality. Linear models with genotype as fixed effect were fitted using SPSS. No significant differences between genotypes were observed (n.s. not significant p>0.05).
(0.11 MB TIF)

Figure S2 A) Mice of the indicated genotypes were injected i.p. with 5×10^6^ *L. monocytogenes* or PBS as a control. After 24 h of infection, mice were killed and spleens isolated. Protein was extracted from spleens of four infected mice per genotype (1–4) or from mice injected with PBS (−), and Western blot for the indicated proteins was performed. STAT1 and STAT2 activation was detected using antibodies recognizing phosphorylated tyrosine 701 and 689, respectively. As a positive control for STAT phosphorylation, Raw264.7 macrophages were infected (+) or not (−) with *L. monocytogenes* for 4 h at a MOI of 10, and protein extracts were loaded onto the same gel. B) BMM derived from mice of the indicated genotypes were infected with *L. monocytogenes* at a MOI of 10. At the indicated time points, protein extracts were prepared and subjected to Western blotting. STAT2 activation was detected using an antibody recognizing phosphorylated tyrosine 689. A+B) For control of equal loading, the blots were reprobed with an antibody specific for the Erk kinases Erk1 and Erk2 (panErk).
(1.23 MB TIF)

Figure S3 A–C) Splenocytes were isolated from C57BL/6, IFNAR1fl/fl-LysMCre, and IFNAR1−/− mice and CD11b+ cells were enriched by magnetic activated cell sorting (MACS). CD11b-enriched cell fraction was stained for CD11c and IFNAR1 and percentages of CD11c+IFNAR+ and CD11c+IFNAR− cells were determined by flow cytometry. D–F) CD11b-enriched splenocytes were stained for CD11b and IFNAR1. IFNAR1 expression by cells gated for coexpression of CD11b was analysed by flow cytometry. G) Peritoneal exudate cells were isolated from wt C57/BL6 mice (solid line), IFNAR1fl/fl-LysMcre mice (dashed line), and IFNAR1−/− mice (gray shaded curve). IFNAR1 expression by cells gated for coexpression of F4/80 (left diagram) was determined by flow cytometry. As a control IFNAR1 expression on total lymphocytes (gated on lymphocytes in FSC/SSC plot (not shown) was analysed.
(1.38 MB TIF)

Figure S4 A) C57BL/6 wt or TLR9−/− mice were injected i.p. with 5×10^6^ *L. monocytogenes* or PBS as a control. After 24 h of infection, mice were killed and spleens isolated. Protein was extracted from spleens of three infected mice per genotype (1–3) or from mice injected with PBS (−), and Western blot for the indicated proteins was performed. STAT1 and STAT2 activation was detected using antibodies recognizing phosphorylated tyrosine 701 and 689, respectively. As a positive control for STAT phosphorylation, Raw264.7 macrophages were infected (+) or not (−) with *L. monocytogenes* for 4 h at a MOI of 10, and protein extracts were loaded onto the same gel. For control of equal loading, the blots were reprobed with an antibody specific for the Erk kinases Erk1 and Erk2 (panErk). B) mDC of the indicated genotypes were infected with *L. monocytogenes* at a MOI of 10. At the indicated time points, total RNA was prepared. The isolated RNA was reverse-transcribed and induction of the IFNβ gene was measured by Real-time PCR. For normalization to a house-keeping gene, GAPDH was measured.
(0.28 MB TIF)

Text S1 Supplementary Materials and Methods
(0.03 MB DOC)
