# Supplementary figures and images for: Characterization of the Interferon-Producing Cell in Mice Infected with Listeria monocytogenes
Source: PLoS Pathog. 2009 Mar 27;5(3):e1000355. doi: 10.1371/journal.ppat.1000355 (PMC2654726; doi:10.1371/journal.ppat.1000355)

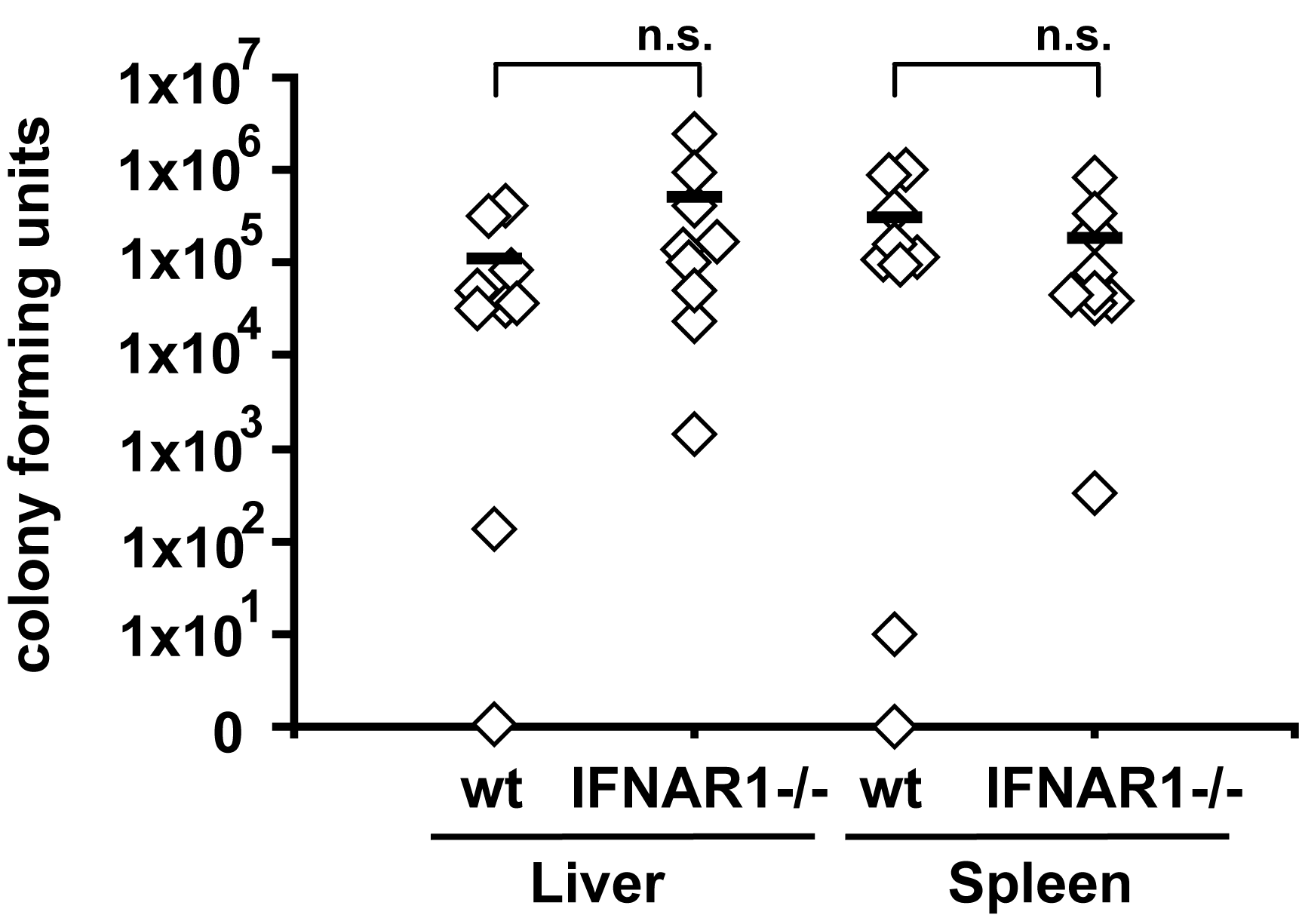

Supplement: Figure S1 — Groups of 9 C57BL/6 wt or IFNAR1−/− mice were infected with 5×10^6^ L. monocytogenes. After 24 h of infection, mice were killed and the L. monocytogenes titre was determined in the liver and spleen and presented as CFU. Data were log transformed to achieve approximate normality. Linear models with genotype as fixed effect were fitted using SPSS. No significant differences between genotypes were observed (n.s. not significant p>0.05). (0.11 MB TIF) [file ppat.1000355.s001.tif]

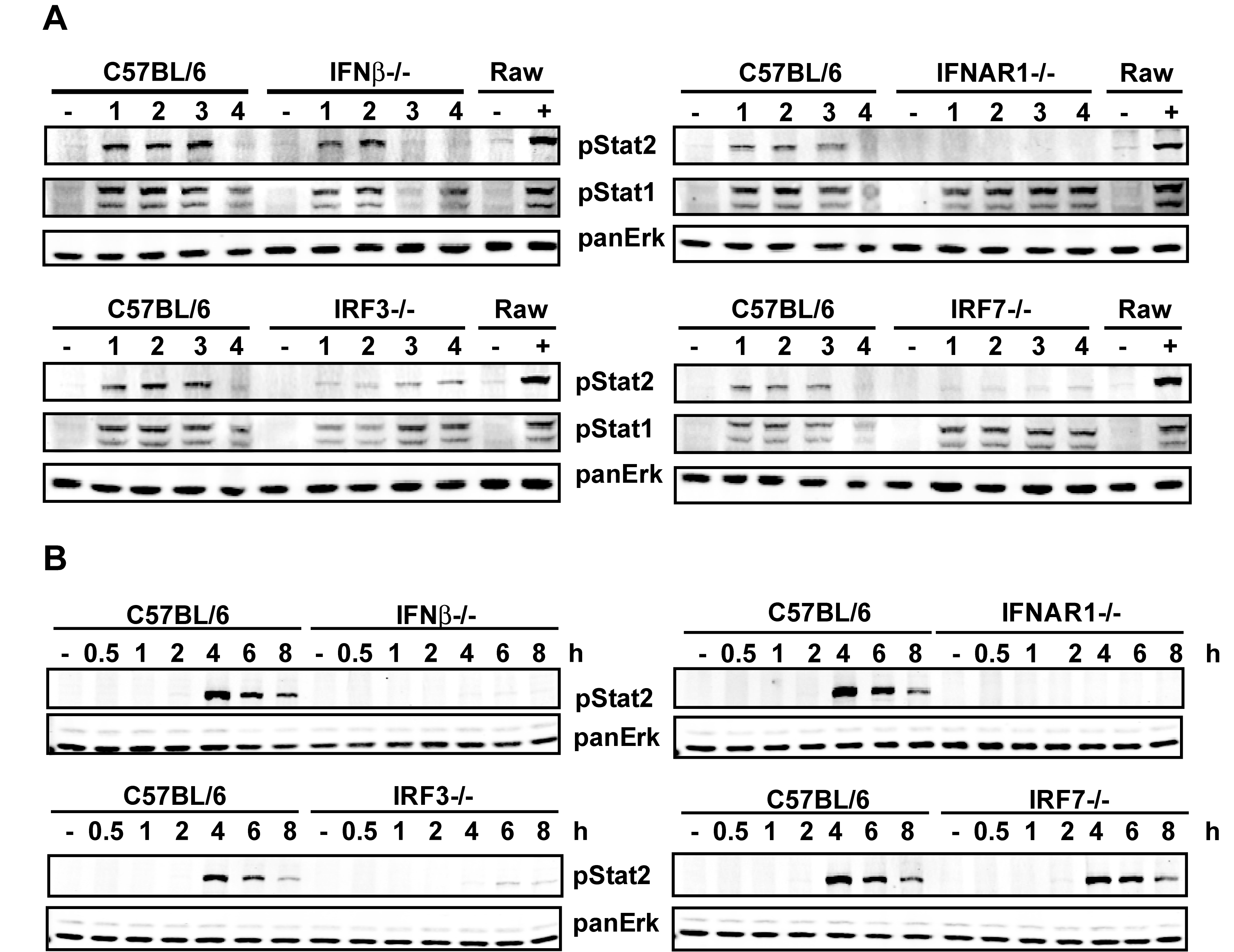

Supplement: Figure S2 — A) Mice of the indicated genotypes were injected i.p. with 5×10^6^ L. monocytogenes or PBS as a control. After 24 h of infection, mice were killed and spleens isolated. Protein was extracted from spleens of four infected mice per genotype (1–4) or from mice injected with PBS (−), and Western blot for the indicated proteins was performed. STAT1 and STAT2 activation was detected using antibodies recognizing phosphorylated tyrosine 701 and 689, respectively. As a positive control for STAT phosphorylation, Raw264.7 macrophages were infected (+) or not (−) with L. monocytogenes for 4 h at a MOI of 10, and protein extracts were loaded onto the same gel. B) BMM derived from mice of the indicated genotypes were infected with L. monocytogenes at a MOI of 10. At the indicated time points, protein extracts were prepared and subjected to Western blotting. STAT2 activation was detected using an antibody recognizing phosphorylated tyrosine 689. A+B) For control of equal loading, the blots were reprobed with an antibody specific for the Erk kinases Erk1 and Erk2 (panErk). (1.23 MB TIF) [file ppat.1000355.s002.tif]

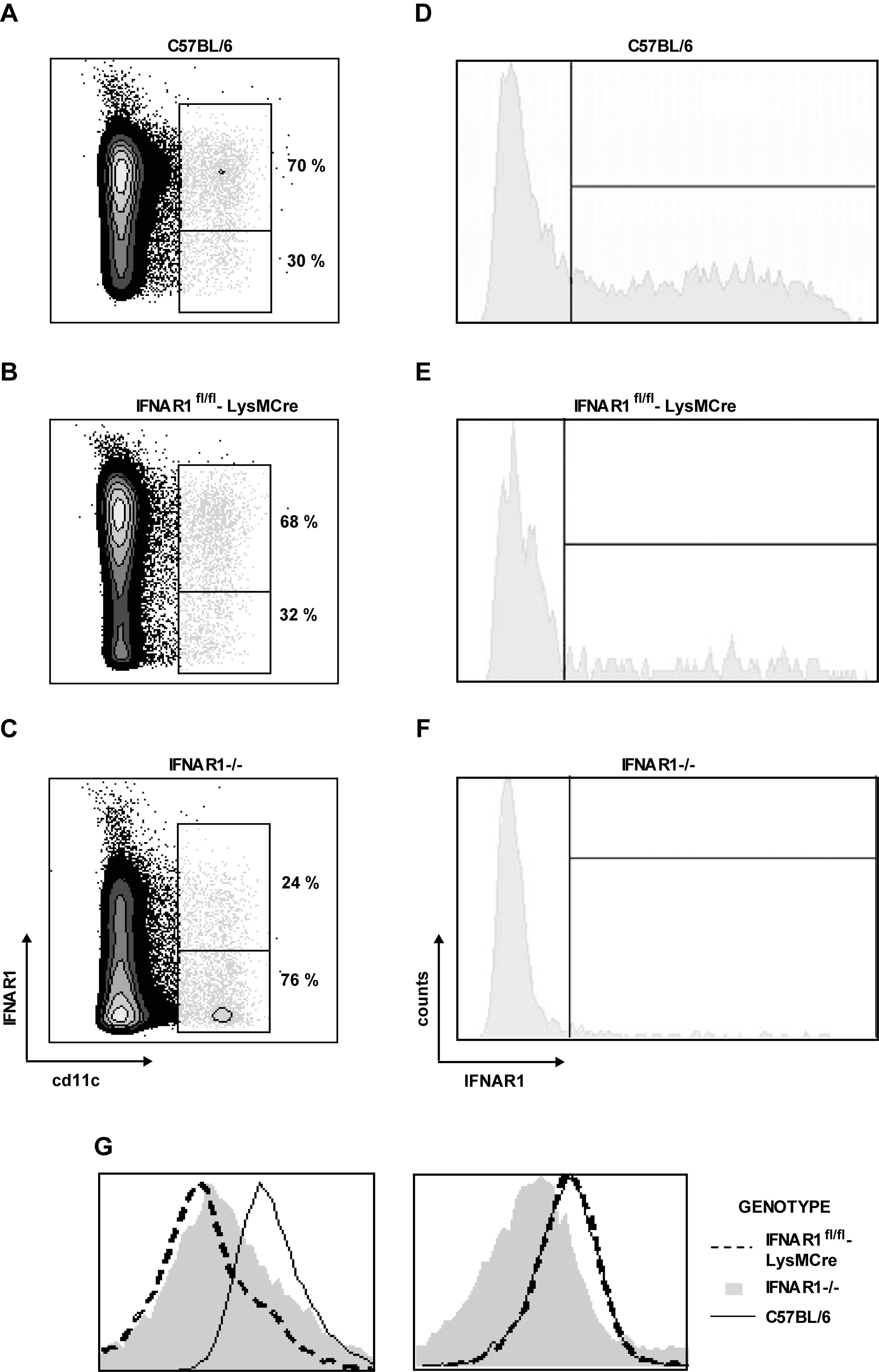

Supplement: Figure S3 — A–C) Splenocytes were isolated from C57BL/6, IFNAR1fl/fl-LysMCre, and IFNAR1−/− mice and CD11b+ cells were enriched by magnetic activated cell sorting (MACS). CD11b-enriched cell fraction was stained for CD11c and IFNAR1 and percentages of CD11c+IFNAR+ and CD11c+IFNAR− cells were determined by flow cytometry. D–F) CD11b-enriched splenocytes were stained for CD11b and IFNAR1. IFNAR1 expression by cells gated for coexpression of CD11b was analysed by flow cytometry. G) Peritoneal exudate cells were isolated from wt C57/BL6 mice (solid line), IFNAR1fl/fl-LysMcre mice (dashed line), and IFNAR1−/− mice (gray shaded curve). IFNAR1 expression by cells gated for coexpression of F4/80 (left diagram) was determined by flow cytometry. As a control IFNAR1 expression on total lymphocytes (gated on lymphocytes in FSC/SSC plot (not shown) was analysed. (1.38 MB TIF) [file ppat.1000355.s003.tif]

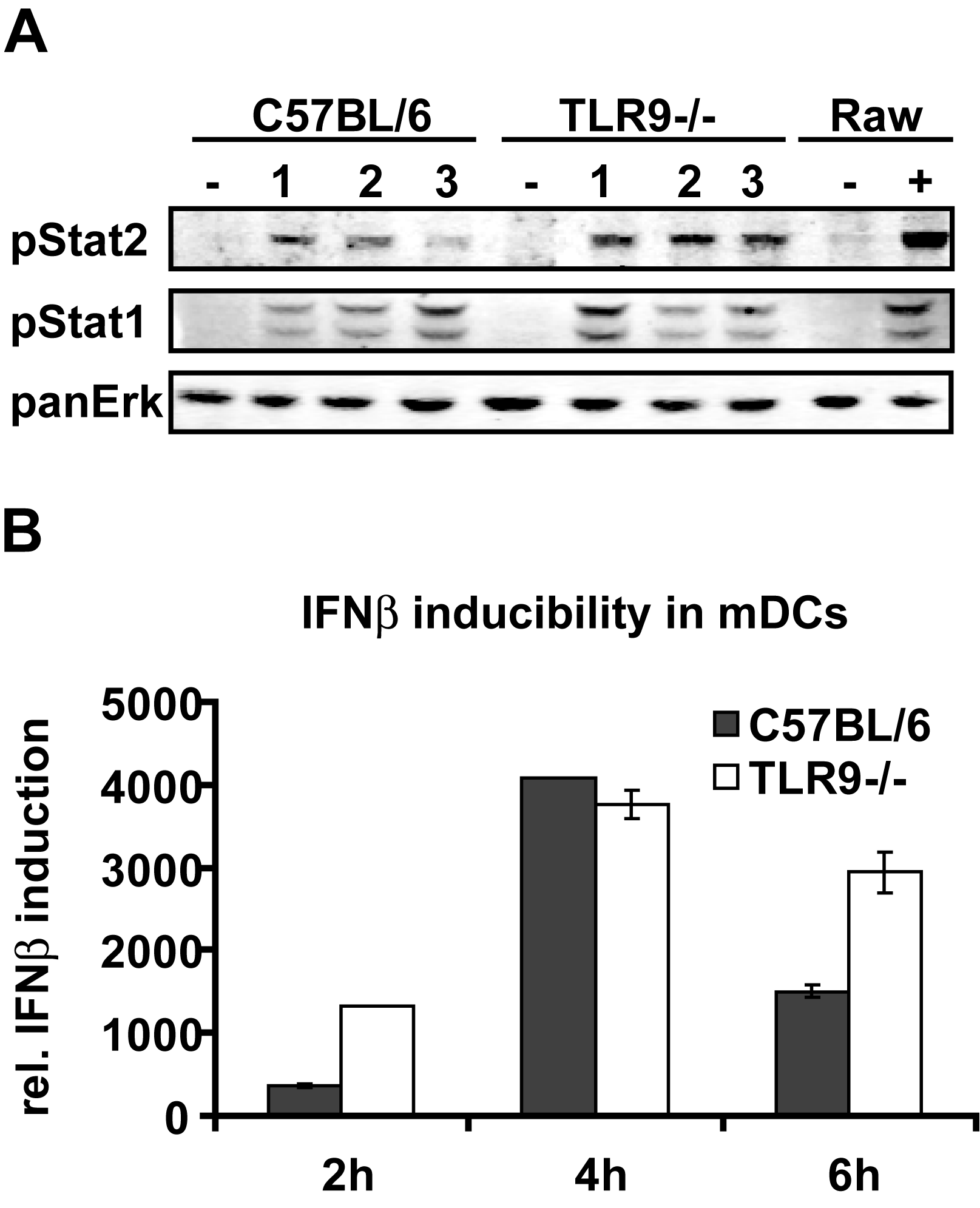

Supplement: Figure S4 — A) C57BL/6 wt or TLR9−/− mice were injected i.p. with 5×10^6^ L. monocytogenes or PBS as a control. After 24 h of infection, mice were killed and spleens isolated. Protein was extracted from spleens of three infected mice per genotype (1–3) or from mice injected with PBS (−), and Western blot for the indicated proteins was performed. STAT1 and STAT2 activation was detected using antibodies recognizing phosphorylated tyrosine 701 and 689, respectively. As a positive control for STAT phosphorylation, Raw264.7 macrophages were infected (+) or not (−) with L. monocytogenes for 4 h at a MOI of 10, and protein extracts were loaded onto the same gel. For control of equal loading, the blots were reprobed with an antibody specific for the Erk kinases Erk1 and Erk2 (panErk). B) mDC of the indicated genotypes were infected with L. monocytogenes at a MOI of 10. At the indicated time points, total RNA was prepared. The isolated RNA was reverse-transcribed and induction of the IFNβ gene was measured by Real-time PCR. For normalization to a house-keeping gene, GAPDH was measured. (0.28 MB TIF) [file ppat.1000355.s004.tif]
