# Supplementary material for: Characterization of the Interferon-Producing Cell in Mice Infected with Listeria monocytogenes
Source: PLoS Pathog. 2009 Mar 27;5(3):e1000355. doi: 10.1371/journal.ppat.1000355 (PMC2654726; doi:10.1371/journal.ppat.1000355)
Supplement: Text S1 — Supplementary Materials and Methods (0.03 MB DOC) [file ppat.1000355.s005.doc]

**Text S1**

### Flow cytometric analysis and cell enrichment. For enrichment of CD11b^+^ cells from total spleen, CD11b microbeads were used along with autoMACS™Pro Separator (Miltenyi Biotech) according to manufacturer’s instructions. For FACS analyses cells were stained with anti-CD11c-PE-Cy5.5 mAb (Caltag), anti-CD11b-FITC mAb (BD Pharmingen), and anti-F4/80-PE mAb (Serotec). IFNAR1 was detected using anti-IFNAR1-biotin mAb (kindly provided by Robert Schreiber) and by streptavidin-APC (BD Pharmingen). FACS experiments were performed on LSRII and analyzed using DIVA 6.1.1 software.

**Isolation of proteins and Western blot**. Protein isolation from cells was performed as described (1). Spleens were isolated from mice infected with *L. monocytogenes* or injected with PBS for 24 h, shock-frozen and stored at -80°C. For protein isolation, 20 mg of frozen tissue was homogenized in 1 ml of lysis buffer (10 mM Tris-HCl pH 7.05, 50 mM NaCl, 30 mM NaPPi, 50 mM NaF, 2 mM EDTA, 1 % Triton X-100, 0.1 mM sodium vanadate, 1 mM phenylmethylsulfonyl fluoride, protease inhibitor cocktail (Roche)) with the Precellys 24 homogenizer (Peqlab) 2 x at 6000 rpm for 30 sec. Extracts were cleared by 3 x centrifugation at 15000 rpm, 4°C for 15 min, mixed with Laemmli buffer and boiled for 10 min. Proteins were resolved by 7.5 % SDS-PAGE, and Western blot was performed as described (1). Primary antibodies were used as already described (2). Antibody recognizing Stat2 phosphorylated on tyrosine 689 was purchased from Upstate and used at a dilution of 1:1000. For analysis, blots were probed with fluorescence-labelled secondary antibodies from Molecular probes (Invitrogen) at a dilution of 1:20000 and detected by the Odyssey infrared imaging system (LI-COR, Lincoln, NE).

**References**

1. Kovarik P, Stoiber D, Novy M, Decker T (1998) Stat1 combines signals derived from IFN-gamma and LPS receptors during macrophage activation. *EMBO J.* 17:3660-3668.

2. Stockinger S, Reutterer B, Schaljo B, Schellack C, Brunner S *et al.* (2004) IFN Regulatory Factor 3-Dependent Induction of Type I IFNs by Intracellular Bacteria Is Mediated by a TLR- and Nod2-Independent Mechanism. *J Immunol* 173:7416-7425.
